# Compliance Level and Stability of Micronutrients in Fortified Maize Flour in Tanzania

**DOI:** 10.1155/2024/7746750

**Published:** 2024-02-22

**Authors:** Abdulsudi Issa-Zacharia, Gudila Boniface Mareni

**Affiliations:** Department of Food Science and Agro-processing, School of Engineering and Technology, Sokoine University of Agriculture, P.O. Box 3006, Chuo Kikuu, Morogoro, Tanzania

## Abstract

Maize flour fortification was introduced in Tanzania in 2011 to address the risk of micronutrient deficiency to children, adolescents, and women of childbearing age. Fortified maize flours are processed by small-scale processors who are exempted from mandatory fortification. The current study is aimed at assessing the compliance and stability of fortified processed maize flour with zinc, iron, and folic acid by small-scale processors in comparison to the recommended Tanzania national standards (TZS 328). A total of 69 samples of fortified maize flour were collected at the point of production and retail outlets in Dar es Salaam and Morogoro municipalities, Tanzania. Micronutrients (zinc and iron) were analysed using microwave plasma atomic emission spectrometry (MP-AES), and folic acid was analysed using high-performance liquid chromatography (HPLC). The mean concentrations of micronutrient were significantly (*p* < 0.05) higher at the production site compared to the retail outlet. The amount of iron, zinc, and folic acid in the samples at the production site was 27.17 ± 1.63 mg/kg, 30.56 ± 2.01 mg/kg, and 0.69 ± 0.02 mg/kg, respectively, while it was 19.34 ± 0.97 mg/kg, 21.71 ± 1.50 mg/kg, and 0.49 ± 0.02 mg/kg for iron, zinc, and folic acid, respectively, at the retail outlets. Only 31.6% of the assessed samples from production and 12.9% from retail outlets complied with the recommended national standard. The stability of iron, zinc, and folic acid for the fortified maize flour stored at room temperature (20-32°C) for six months was 95.8%, 96.9%, and 66.9%, respectively. Further investigation on the consistency performance of the dosifier and consistency training of working in the processing unit on the requirements of fortification standards should be done.

## 1. Introduction

Vitamins and minerals are the micronutrients that are required by the body in small quantities and play an important role in the body's normal growth and development. Deficiency of these micronutrients in combination with others causes several problems including stunting growth in children, reduced cognitive function, anemia, neural tube defect, and blindness [1–3]. It also increases the rate of mortality and morbidity especially in children and women of reproductive age [4–6]. Micronutrient deficiency in Tanzania is still challenging, especially in children and women of reproductive age. The Tanzania demographic survey report indicated that 45% of reproductive-aged women [1–3, 6–37] are anemic [33] while 30% had iron deficiency and 35% of children had iron deficiency with high prevalence in children aged 6-11years and 12–23 months [22]. Fifty-eight percent of children aged 6 to 59 months had iron deficiency with anemia. The rate of stunting is 34% and vitamin A deficiency in children aged 0-59 months is 33% while 22% of children are underweight [33].

Food fortification programs have been implemented to help reduce the prevalence of such micronutrient deficiencies [38]. Food fortification programs were recommended by WHO as the main strategy and the same have been adopted by many countries including African countries [12]. Such programs include salt iodization, milk fortification with vitamin D and calcium, and vitamin D fortification of juices. Diseases that were a worldwide problem in the early 20th century such as iodine deficiency disorders, rickets, beriberi, and pellagra have been significantly reduced following food fortification [39]. In 2011, the Tanzanian government mandated the fortification of vegetable oil, maize, and wheat flour due to increase in micronutrient deficiencies [16]. The vegetable oil is fortified with vitamin A, and wheat and maize flours are fortified with multiple micronutrients such as zinc, iron, folic acids, and vitamin B12 [28]. This mandatory fortification was for large-scale producers leaving behind small-scale processors [28].

Maize (Zea mays L.) is a type of cereal produced worldwide and mostly used for human consumption, feed for livestock, and raw material for industry [10]. About 3.5 million small farmers cultivated maize, and most of the families depend on it as the main meal which makes it to be an important crop in Tanzania as well as staple crops [35, 40]. Naturally, whole maize contains different micronutrients like phosphorus, calcium, potassium, iron, zinc, folate, riboflavin, thiamine, and niacin of which iron accounts for 27 mg/kg, zinc is 22.1 mg/kg, and folate accounts for 0.19 mg/kg [31]. In Tanzania, maize is produced at an average of 6711 tons per year [7] and consumption accounts for 1164 kcal/d which is about 50-80% of the total energy [14].

There are a large number of small-scale millers of maize flour, around 95% being located in urban and village which feed majority of people in the surrounding area [41]. Most of the consumed fortified maize flour circulated in Tanzanian market is processed by small-scale processors who are exempted from mandatory fortification scheme. In Tanzania, there are two certification schemes (compulsory and mandatory certification scheme). Fortification scheme is mandatory, but small-scale processors of fortified maize flours are exempted from this mandatory certification scheme. These small-scale processors fortified maize flour using Sanku dosifier technology installed in the milling machine which automatically dispenses premix during milling. However, most of these small-scale processors do not have laboratory facilities for performing quality assurance at the mill to ensure that the fortified maize flour meets the requirements of the standard [42]. The compliance of fortified food in low- or middle-income countries (LMIC) including Tanzania was found to be around 40%, whereby noncompliance foods were found labelled as compliant which misled consumers on micronutrient contents of the food [27]. So far, there is minimal or no reported information regarding compliance of fortified maize flour processed by small-scale processors. Furthermore, the impact of the fortification program is measured by consistency supply of the desired nutrients at point of consumption [8]. Micronutrients in the fortified food may be accelerated by storage and handling condition of the final product, packing materials, and length of storage [43]. It is the requirement of the standard that premix and fortificant used during maize flour fortification should have stability. For example, it is required that no more than 20% of its origin activity will be lost when stored for 21 days at 45°C in well-closed container at the level of 2.5 g/kg in a milled maize product having moisture in the range of 13.5 to 14.5% [32]. Fortification is done to ensure that the fortified foods reach the targeted group with the required number of micronutrients. This study is aimed at assessing the compliance and stability of fortified maize flour processed by different small-scale processors in Morogoro and Dar es Salaam regions, Tanzania (Mnenuka Grain Mills, Ngange Mills, Msouth Extra Power B M Murua, Sozi Integrity, Isanga Supper Sembe Mohamed Mkasa mills, and Asante Mungu). Also, the study is aimed at providing awareness to the regulatory authority on the compliance of status of fortified maize to the required standard a (TZS 328:2018-EAS 768:2013) and will help them to put effort towards improvement of compliance.

## 2. Materials and Methods

### 2.1. Study Design

The study was conducted at Ubungo in Dar es Salaam and Morogoro municipalities, Tanzania. Samples were collected at production area from small-scale processors who are under fortification program and were under operation approach during the research period.

Cross-sectional design both quantitative and qualitative was used in this study, whereby data were collected at a specific point in time. Collections of data were based on fortified samples that were collected from processors at production line and retail outlets. The fresh samples from the production were stored at room temperature (20-32°C), the same condition that was used to store maize flour by processors. The stability of micronutrients was studied for a period of six months at interval of three months. Semistructured questionnaires were also used to collect information on challenges faced by small-scale processors to attain compliance to the recommended standard.

### 2.2. Sampling and Sample Size

Morogoro municipality in Morogoro region and Ubungo district in Dar es Salaam region were purposively selected based on the availability of high numbers of processors of fortified maize flour. Triplicate samples of 1 kg each from the same batch were collected at production area, after which samples were mixed up to get homogenized composite and about 100 g was kept in air tight aluminium polyethylene bags labelled with unique code number, stored, and transported to TBS laboratory for analysis.

In the retail outlets, a stratified multistage sampling was employed [17]. In the first stage, ten wards of Morogoro municipality, namely, Mwembesongo, Kihonda, Kingolwira, Bigwa, Kiwanja cha Ndege, Mazimbu, Mji Mpya, Kingo, Sabasaba, and Mafiga, were randomly selected from the list of 29 wards. In the second stage, one subward was then purposively selected from each ward based on the volume of trade as advised by ward executive officers. In Ubungo district, 5 wards, namely, Manzese, Mbezi, Sinza, Kibamba, and Kimara, were randomly selected from the list of 14 wards. Second one subward was purposively selected from each ward based on the volume of trade as advised by ward executive officers. Finally, for each small-scale processors visited, four samples of the same brand were randomly purchased from different retail shops located in these subwards. Samples of the same brand were mixed up to get one representative homogenized sample for each processor, and a total of 20 brands from Morogoro municipal and 11 brands from Ubungo municipal were identified. Therefore, 38 brands of fortified maize flour samples were collected from production sites and 31 from retail outlets making a total of 69 samples. Fresh samples from 8 processors were also collected from production areas and stored at room temperature, and the stability of added micronutrients was studied for a period of six months at an interval of three months.

### 2.3. Sample Handling

About 100 g of the composite sample from each processor and the retail outlet was kept in airtight aluminium polyethylene bags labelled with a unique code number and transported to the TBS laboratory. The samples for folic acid analysis were stored in a freezer at -3°C until analysis to prevent loss of vitamins while those for mineral analysis were stored at room temperature (20-32°C). Fortified maize flour from 8 processors was sampled and kept in polypropylene woven bags which are used to pack commercial fortified maize flour. The samples were stored at room temperature (20-32°C), to mimic the storage condition used by processors. The stability of micronutrients was accessed for six months at an interval of three months. Sample information such as sampling date, brand, manufacturer's name, address, sampling source, and identification code number was recorded for easy identification of the sample.

### 2.4. Determination of Iron and Zinc

Sample analysis for iron and zinc was customized by Zhao et al. [36] during which 0.5 g of fortified maize flour was weighed (in triplicate) using analytical balance and transferred into individual Teflon microwave digestion vessels. 5 mL of 32% NHO_3_ and 1 mL of 30% H_2_O_2_ were added to the contents in the flask, and blank containing the same quantity of reagents without addition of sample was also prepared. The flasks containing samples were transferred into an advanced closed microwave system with the maximum allowed temperature and pressure of 150°C and 100 bar, respectively, for digestion. In the digestion chamber, samples were heated up to 150°C from room temperature for 15 minutes. These values were maintained for another 30 min to ensure complete digestion. After digestion, samples were cooled to the ambient temperature of about 30°C for another 20 min before handling. The digested samples were transferred into a 50 mL Teflon tube, labelled, and made up to 50 mL with deionized water read for metal analysis. The concentrations of zinc and iron in the sample were calculated according to AOAC 984.27 method. The % recovery and repeatability of the analysis of the sample spiked with 9 mg/kg were used to evaluate the precision and accuracy of the iron and zinc results. Each measurement was done in triplicate, and the % recovery was calculated.

### 2.5. Determination of Folic Acid

Determination of folic acid was done according to Alaburda et al. [44] with slight modifications using reverse-phase HPLC (Shimadzu LC-20A, Japan). About 5 g of the sample was accurately weighed into centrifuge tubes in triplicate and labelled. Approximately 20 mL of acidified deionized water was added followed by vortexing (IWAKI mixer, model-TM-151, no. 68130, Japan) for 2 minutes. The mixture was then centrifuged (Hettich Zentrifugen, D-78532, Tuttlingen, Germany) at 10000 rpm for 10 minutes. The supernatant was carefully removed and filtered through a 0.45 *μ*m Chromafil® Xtra MV-45/25 disposable syringe and kept in 2 mL vial for HPLC analysis. Analysis of the samples was done using reverse-phase HPLC (Shimadzu LC-20A, Japan). The chromatogram separation of folic acid was attained at 4.8 min at 284 nm. Quality assurance was done by spiking the sample with 2 mg/kg of folic acid standard (analytical grade batch no. 9.0 from France to Europe), and the recovery was calculated.

### 2.6. Determination of Moisture Content

The moisture content of fortified maize flour was determined using oven drying method [45] as described by Nielsen and Bradley [23]. Approximately 10 g of the sample was weighed in an already dried and cooled aluminium dish and evenly distributed. The aluminium dish was placed in an air-dry oven (HERAEUS) at 105°C and dried for 3 hours. The dishes were covered and placed in a desiccator for 30 min to reach the ambient temperature before being weighed. The loss in weight was calculated as the moisture content of the fortified maize flour.

### 2.7. Statistical Analysis

Analysis of the samples was done in triplicate. In each case, the mean (mg/kg) and the standard deviation were calculated using R software version 3.5 (2020). A two-way analysis of variance (ANOVA) was used to test the significance of difference on the level of iron, zinc, and folic acid among the production and retail outlet fortified maize samples and the effect of storage condition and a *p* value *<* 0.05 being considered statistically significant. This means separation was done by Turkey's honest significant difference (HSD). Compliance of micronutrients in the fortified maize flour against the recommended national standard was tested using Statistical Package for Social Sciences (IBM SPSS® Version 25, 2017). Frequencies were computed to determine challenges faced by processors in achieving compliance with the recommended standards. A chi-square was computed to assess if there is any association between compliance of fortified maize flour and the sample source.

## 3. Results and Discussion

### 3.1. Level of Micronutrients in the Fortified Maize Flour

The mean concentrations of the micronutrient analysed are reported in Table 1. The concentration of iron, zinc, and folic acid from production sites was significantly higher than that of retail outlets at *p* < 0.05. The mean concentrations of iron, zinc, and folic acid from production sites were 27.17, 30.56, and 0.69 mg/kg, respectively. It ranged from 5.79 to 77.66, 5.62 to 118.15, and 0.29 to 1.47 mg/kg for iron, zinc, and folic acid, respectively (Table 1). In the retail outlet samples, the mean concentrations of iron, zinc, and folic acid were 19.34, 21.71, and 0.49 mg/kg, respectively, and their value ranged from 6.15 to 35.81, 2.67 to 46.69, and 0.22 to 1.12 mg/kg for iron, zinc and folic acid, respectively.

Although all processing facilities visited used the Sanku dosifier technology to fortify maize flour and the same premix was supplied by one supplier, the level of micronutrients obtained in the samples collected varied from one processor to another. The obtained mean concentrations of micronutrients in fortified maize flour appeared to be statistically different at *p* < 0.05 among production sites and retail outlet samples as shown in Table 1. This could be due to inadequate mixing of the maize flour during fortification. The study in Cameroon reported a different and low level of micronutrients in fortified wheat flour for the market samples compared to the industry samples and concluded that inadequate mixing of the product during fortification was the major cause [20]. Moreover, the lack of internal monitoring of fortified maize flour as observed during processing can be the cause of the inconsistency level of micronutrients observed in the current study. It was observed during the survey that only 2.6% of processors had a capacity to conduct qualitative analysis of the fortified maize flour at the factory. The findings observed in the current study were similar to postmarket surveillance conducted by TFDA on wheat flour which revealed inadequate quality control at the factory as a factor that contributes to inconsistency micronutrients [24]. In another study, Enzama et al. [42] reported that quality control facilities were a problem in most African countries, and fortification was not owned by the small mills as they considered undertaking quality control at the mill as the additional costs which was also contributed by lack of knowledge on quality control and quality assurance.

### 3.2. Compliance of Fortified Maize Flour with the Recommended Standard

Results on compliance of fortified maize flour to the recommended standard are presented in Table 2. According to Tanzanian standards of fortified maize flour (TZS 328/EAS 768), the recommended amount of fortified maize flour is 31 ± 10 mg/kg for iron, 49 ± 16 mg/kg for zinc, and for folic acid 1.2 ± 0.5 mg/kg at the factory and 0.6 mg/kg to 1.7 mg/kg at regulatory level [32]. The specific micronutrients analysed (iron, zinc, and folic acid) in the flour samples were out of the range specified in the TZS 328 for fortified flour. More than half of the samples from the production site (68.4%) did not comply with the recommended standard while at retail outlet point, more than 87.1% did not comply. The observed low level of compliance of micronutrients in fortified maize flour will not solve the aim of the government of fortifying maize flour to alleviate micronutrients deficiencies in the society. Chi-square analysis shows that there is no significant association between compliance and source of samples at *p* > 0.05 although the compliance at production tended to be higher than that of retail outlet.

**Figure 1:**
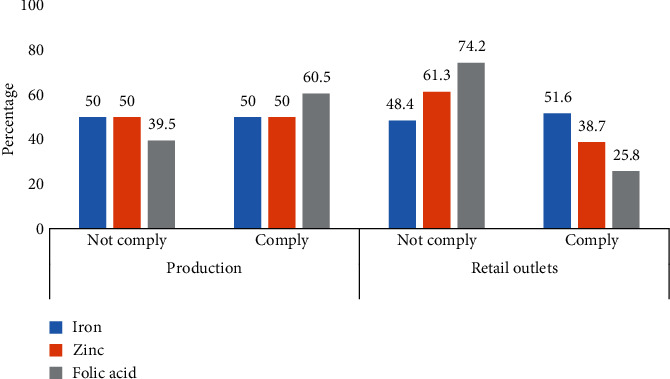
Compliance of specific micronutrient with the recommended standard. Compliance: iron, 31 ± 10 mg/kg; zinc, 49 ± 16 mg/kg; and folic acid, 1.2 ± 0.5 mg/kg. Noncompliance: iron, <21 mg/kg > 41 mg/kg; zinc, <33 mg/kg > 65 mg/kg; and folic acid, <0.6 mg/kg > 1.7 mg/kg. Tanzania national standards (TZS328).

The results as shown in Figure 1 indicated that about 50% of the collected samples at the production area complied with the recommended standard for iron and zinc while 60.5% complied for folic acid. On the other hand, 51.6%, 38.7%, and 25.8% of samples collected from the retail outlet complied with recommended standards for iron, zinc, and folic acid, respectively.

**Figure 2:**
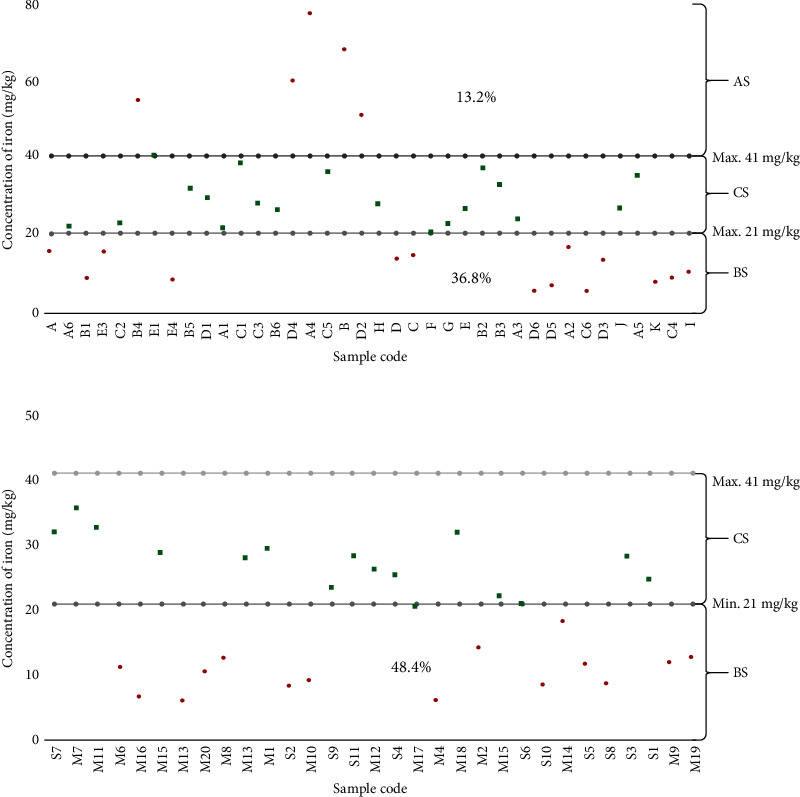
Mean distribution of iron concentration for the composite samples of fortified maize flour. The iron content of fortified maize flour for production samples (a). Iron contents of fortified maize flour samples from retail outlets (b). Samples that lie above the recommended level (AS). The sample that lies below the recommended level (BS). Compliance samples to the recommended standard (CS). All samples were measured in triplicate. Samples were coded with a unique identified number for the purpose of hiding the identity. Sample coded with letter S are collected from selling outlets in Dar es Salaam region while samples coded M are selling outlet from Morogoro region. Samples coded A to K are collected from production line in Dar es Salaam region while letter A1 to E4 are production samples from Morogoro region.

Further analysis was carried out to compare the compliance of specific micronutrients at production and retail outlets. There was no statistical significance between compliance of iron at production and those of retail outlets (*χ*^2^ = 0.018, *p* = 0.8). Although there was no significant difference, nevertheless, iron concentration at retail outlets tended to be higher than those of production. Moreover, five (5) samples (13.2%) from the production site were above the recommended standard, while 14 samples (36.8%) were below the recommended standard. In the retail outlets, 48.4% of the samples were below the recommended level and none of them exceeded the recommended level (Figure 2).

The obtained compliance level in iron observed in the current study (50% from production site and 51.6% from retail outlets) was higher than those reported in Nigeria for the fortified maize flour from factories and markets in which only 18.2% complied with the recommended Nigerian standards [26]. Moreover, the obtained low level of compliance in iron could be due to lack of internal monitoring of the product during fortification and insufficient addition of micronutrients at the factory level. Similarly, the study conducted in South Africa reported the insufficient addition of micronutrients at the factory as the major cause of low level of compliance in fortified flour [40]. Uncontrolled fortification practices at factories can also lead to excessive or inadequate levels of micronutrients in the finished products which put in danger the health of consumers if the dose rich toxic level or the amount of consumed nutrients is ineffective [34].

**Figure 3:**
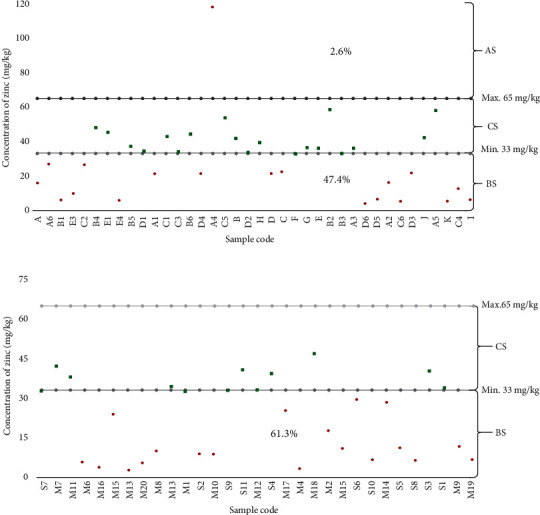
Mean distribution of zinc concentration for the composite samples of fortified maize flour. Zinc content of fortified maize flour samples from production (a). Zinc contents of fortified maize flour samples from retail outlets (b). Samples that lie above the recommended level (AS). The sample that lies below the recommended level (BS). Compliance samples to the recommended standard (CS). All samples were measured in triplicate.

Further analysis was carried out for zinc, and the results indicated high compliance in samples from the production site as compared to that of the retail outlet; nevertheless, the level of compliance was not significant at *p* = 0.34. About half of the samples at the production site (47.4%) were below the recommended level, and 2.6% exceeded the recommended level, while 61.3% of the retail outlet's samples were below the recommended level (Figure 3).

Fortification is aimed at ensuring that the right and correct amount of micronutrients reaches the targeted group of people to prevent certain health condition without exceeding or lowering the recommended level of micronutrients [37]. The level of zinc reported in the current study (Figure 3) was higher than the one reported in a study carried out in Kenya by Khamila et al. [15] in which only 22.2% of zinc in fortified maize flour was below the recommended Kenyan standard. The observed variation of the results could be attributed to inconsistency dozing and mixing of micronutrients by dosifier during fortification. The study by Yusufali et al. [40] in South Africa argued that the insufficient addition of micronutrients at the factory level causes a great variation of micronutrients in fortified food.

**Figure 4:**
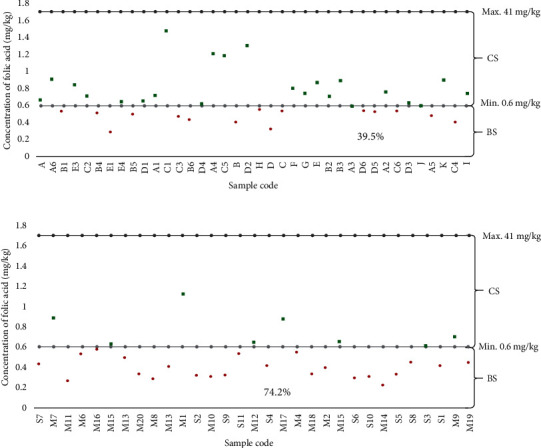
Mean distribution of folic acid concentration for the composite samples of fortified maize flour. Folic acid content of fortified maize flour samples from production **(**a). Folic acid contents of fortified maize flour samples from retail outlets (b). Sample that lies below the recommended standard (BS). Compliance samples to the recommended standard (CS). All samples were measured in triplicate.

The analysis of folic acid in fortified maize flour for the samples collected indicated that the compliance of folic acid at retail outlets was significantly lower than that of production at *p* = 0.0002. Also, more than one-third (39.5%) of the samples at the production site and 74.2% of retail outlets samples were below the recommended standards (Figure 4).

The fortified maize flour analysed showed folic acid levels ranging from 0.22 mg/kg to 1.47 mg/kg. The compliance of samples from retail outlets (25.8%) was almost the same as the one reported in Kenya (29.6%) in the fortified maize flour collected from the market. Also, the obtained low level of compliance in the current study (Figure 4) was higher than those reported by Khamila et al. [15] in fortified maize flour in which only 11.1% was below the detectable limit. The higher level of noncompliance (below the recommended standard) observed in the current study could be attributed to inadequate mixing of products during fortification and improper handling and storage of the product in the market. Combining the ingredients of a fortificant premix including micronutrients (vitamins and/or minerals) and other ingredients (e.g., antioxidants, additives, and stabilizers) simplifies the addition of the fortificants to the maize flour during production and improves accuracy in the mixing and distribution of these micronutrients throughout the maize flour. During the survey, the fortified maize flour was found exposed directly to sunlight and others were kept open in the air which resulted in the loss of vitamin. Vitamins such as vitamin B_9_ (folic acid), vitamin A, and vitamin B_2_ (riboflavin) are sensitive to heat, oxygen, light, and acidic environments. The current study was conducted in similar conditions, and these could be a possible reason for the low retention of such vitamins. A previous study reported that in such conditions, losses could be up to 50-90% for folate, 70-95% for retinol, and 78% for riboflavin [46]. This was supported by the study conducted in Brazil on fortified corn and wheat flour which reported that inadequate homogenization of vitamin at the processing stage causes a low level of micronutrients [47]. Moreover, vitamins are susceptible to deterioration when exposed to unfavorable environments such as light, temperature, relative humidity, and oxygen [18, 46]. It was recommended by Luthringer et al. [19] that poor storage conditions result in reduction of the retention capacity of vitamins which can lead to noncompliance of the fortified product.

Despite the initiatives taken by the Tanzania government to control diseases related to micronutrient deficiencies, compliance of fortified maize flour to the recommended Tanzanian standard is still a challenge. This can be justified by the noncompliance of 76.8% for the total samples collected from production and retail outlets which was higher than the findings by Ogunmoyela et al. [26] who reported that only 45% of maize and wheat flour, sugar, and vegetable oil for the samples collected from the factory complied with the recommended Nigerian standard.

Generally, the observed low level of compliance in fortified maize flour in the current study could be attributed to operations of small-scale processors of fortified maize flour in Tanzania on voluntary basis in which they are not bound by the laws and regulations in making sure the final products are adequately fortified. A food fortification program was desired to control micronutrient deficiency through the delivery of quality and safe food to the targeted group of people. Consumers bought products relying on manufacturers' claims on labelling regarding nutritional quality information, and they can be misled by manufacturers when operation is under voluntary and food control agencies have not been powered to enforce the standard [12, 21]. Voluntary fortification was introduced in Tanzania to small-scale processors to increase the coverage of fortified food since most maize flour is produced by small-scale processors. Zimmerman et al. [37] found that the coverage of iodized salts increased from 40% to 49% in the countries which operated under voluntary fortification compared to 49% to 72% for countries that operated under mandatory fortification. Voluntary fortification of maize flour could weaken the fortification program if not well monitored leaving targeted people consuming nonfortified, under-/overfortified food [21] due to challenges associated with consistency fortification of the product with an adequate amount of micronutrients [48].

### 3.3. Stability of Micronutrients (Iron, Zinc, and Folic Acid) of Fortified Maize Flour Stored under Room Temperature

Table 3 shows the effect of storage time on the level of micronutrients in fortified maize flour. Both zinc and iron assessed were within the limit of the recommended standard in all six months of storage. The statistical analysis indicated that storage time had no effect on the level of zinc content for all six months of storage. Although generally there was no significant change in iron concentration for the first three months of storage, the significance decreases at *p* > 0.05 was observed at the end of six month. Folic acid decreased significantly to all six months of storage. Moreover, a significant increase in moisture was observed in all months of storage. The decrease in folic acid could be due to the instability of folic acid in the fortified maize flour influenced by high temperature, light, and moisture contents. Folic acid is a water-soluble vitamin, and high moisture contents accelerate its solubility.

The efficacy of fortification is measured by ensuring that the fortified food reaches the targeted group of people with the desired level of micronutrients [8], and this depends on the stability of micronutrients added in food under various conditions. The analysis from this study indicated that the stability of iron and zinc in fortified maize flour stored at room temperature for six months was more than 95% with only 4.2% and 3.1% loss in iron and zinc, respectively (Table 4). Statistically, there was a significant change in iron at the end of six months which might be due to the solubility of sodium iron ethylenediaminetetraacetic acid (NaFe-EDTA) in water [9, 13] as during storage, moisture contents were observed to increase significantly for all six months of storage (Table 4). The increase of moisture contents could be due to the permissibility of moisture into polypropylene bags during storage. The stability of minerals in fortified flour stored at room temperature is supported by the study conducted by Rosado et al. [2] in which more than 95% of iron was retained in corn flour stored for 90 days with no significant change in zinc content. Nuzhat [25] also reported 97-100% of iron stability in wheat flour. Moreover, high stability of more than 90% of iron contents was also reported by Kuong et al. [18] in the fortified cold extruded rice stored for 12 months. The observed results in the current study could be attributed to the stability of minerals added to fortified maize flour [15]. Research has shown that minerals are more stable than vitamins and thus have a higher retention capacity as can be observed in the current study. Hemery et al. [11] reported that iron and zinc have high stability during storage at high temperatures (40°C) and high humidity (75%) for 12 months as compared to losses of up to 90% at the highest temperature and humidity for vitamins to further confirm that minerals are more stable than vitamins on exposure of different conditions such as light, heat, and temperature. The superior rates of iron retention in the case of NaFe-EDTA-fortified product could be attributed to its highly stable structure, which resists the leaching of iron from the EDTA structure [30]. The observed % loss of iron 1.3% and zinc 2.1% in the present study at the end of three months was less than the 14% loss in iron reported by Rosado et al. [2] in fortified pasta stored at room temperature for two months and 4.39% loss in zinc reported by Akhtar et al. [49] in chapattis made by fortified wheat flour stored for two months at room temperature.

The stability of folic acid added in fortified maize flour was higher in the first three months 78.3% and decreased to 66% at the end of six months. In the current study, the percentage loss of folic acid was 21.7% at the end of three months and increased significantly to 33% at the end of six months. The decrease in stability of folic acid in the fortified maize flour could be influenced by the significant increase in moisture contents as observed in all six months of storage (Table 4). A similar loss was reported by Khamila et al. [15] who observed the highest proportion of folic acid loss of 19.3% in the fifth and 24.1% in the sixth month for the samples stored at RH 75%. A previous study conducted in Brazil observed a significant decrease in folic acid for the corn flour samples which were stored at room temperature [47]. Also, the study conducted by Hemery et al. [11] concluded high losses of folic acid in the first three months for the fortified flour samples stored in paper bags at 25-40°C. Losses of folic acid observed in the current study could be associated with the instability of folic acid in high temperature, light, and moisture. The previous study supports this argument and reported that folic acid is unstable and loses activity in the presence of heat, light, oxidizing agents or reducing agents, and acidic or alkaline environments [50]. Folic acid is a water-soluble vitamin, and high moisture contents accelerate its solubility. Dunn et al. [46] have reported that both packaging materials, moisture contents, and duration storage time have affected micronutrients in fortified food.

## 4. Conclusions

The study indicated a low level of compliance for the fortified maize flour to the recommended Tanzania national standard for both production and retail outlet samples. The most probable reasons for the observed noncompliance are the inconsistency and underfortification of micronutrients at the factory level due to a lack of technical knowledge on internal monitoring of the final product at the factory during processing which makes the product to be released to the market with unknown quality. Furthermore, improper storage of premix and fortified maize flour could be the cause of low compliance especially in folic acid which is highly affected by sunlight and excessive heat. Since small-scale processors are not required by laws and regulations to ensure the product is adequately fortified, their operation on a voluntary basis has resulted in a poor degree of compliance. In addition, the stability of the micronutrients in fortified maize flour was greater than 95% for minerals (zinc and iron) and 66% for folic acid when stored at room temperature for six months in polypropylene bags. For proper implementation of the fortification program and to ensure that the recommended quantity of micronutrients reaches the targeted people, it is recommended that an investigation on the performance of dosifier be conducted. To retain folic acid in fortified maize flour, the government should encourage the processors of fortified maize flour to pack the product in hermetic bags which does not allow penetration of moisture hence loss of folic acid. The government is recommended to extend training on fortification required standards to all employees working in the factory regardless of their positions and strengthen monitoring mechanisms of the fortified maize flour to ensure that it is adequately fortified.

## Figures and Tables

**Table 1:** Average ± SE and range for concentration (mg/kg) of micronutrients in maize flour sample at production and retail outlets.

Source of samples	*N*	Micronutrient concentrations (mg/kg)			Moisture contents
		Iron^1^	Zinc^1^	Folic acid^1^	
Production sites (PS)	38	27.17 ± 1.627^a^	30.56 ± 2.013^a^	0.69 ± 0.024^a^	12.57 ± 0.098^a^
Retail outlet (RO)	31	19.34 ± 0.968^b^	21.71 ± 1.498^b^	0.49 ± 0.021^b^	12.59 ± 0.100^a^
Range for PS		5.79-77.66	5.62-118.15	0.29-1.47	11.02-14.65
Range for RO		6.15-35.81	2.67-46.69	0.22-1.12	11.28-14.50

^1^Values are the mean concentration of three replicates times *N* for each sample, and values having different superscripts are significantly different at *p* < 0.05. *N* is the number of samples.

**Table 2:** Compliance of fortified maize flour as compared to the recommended standard grouped by source of samples.

Source of samples	Number of samples	Complied samples	% sample complied	No. of samples not complied	% sample not complied	*χ* ^2^	*p* value
Production	38	12	31.6	26	68.4	3.343	0.07
Retail outlet	31	4	12.9	27	87.1		

The recommended standard values for compliance: iron, 31 ± 10 mg/kg; zinc, 49 ± 16 mg/kg; and folic acid, 1.2 ± 0.5 mg/kg. Noncompliance: iron, <21 mg/kg > 41 mg/kg; zinc, <33 mg/kg > 65 mg/kg; and folic acid, <0.6 mg/kg > 1.7 mg/kg. Tanzania national standards (TZS 328).

**Table 3:** Mean of iron, zinc, and folic acid in fortified maize flour stored at room temperature.

Storage time	Micronutrient content (mg/kg)			% moisture contents^1^
	Iron^1^	Zinc^1^	Folic acid^1^	
Month 0	31.07 ± 1.311^a^	39.09 ± 2.331^a^	0.89 ± 0.059^a^	12.87 ± 0.102^a^
Month 3	30.65 ± 1.306^a^	38.27 ± 2.380^a^	0.70 ± 0.040^b^	13.50 ± 0.067^b^
Month 6	29.74 ± 1.346^b^	37.87 ± 2.378^a^	0.60 ± 0.040^c^	13.78 ± 0.055^c^

The superscript values followed by the same letter in the column are not significant at *p* > 0.05. ^1^Data are the results of mean and SE of eight samples stored at room temperature (20°C-32°C) measured in triplicate.

**Table 4:** Stability of micronutrients in fortified maize flour.

Micronutrients	Stability in 3 months (%)	Stability in 6 months (%)	Loss in 3 months (%)	Loss in 6 months (%)
Iron	98.7	95.8	1.3	4.2
Zinc	97.9	96.9	2.1	3.1
Folic acid	78.3	66.9	21.7	33

Stability of micronutrients (%) in fortified maize flour during 6 months for the samples stored at room temperature. Loss (%) = ((initial concentration of micronutrient − final concentration of micronutrient)/initial concentration of micronutrient) × 100; stability (%) = (final concentration of micronutrient/initial concentration) × 100.

## Data Availability

The master thesis data used to support the findings of this study have been deposited in the Sokoine University of Agriculture repository (SUA IR). These data are also included in the article.
